# dopOSCCI: A functional transcranial Doppler ultrasonography summary suite for the assessment of cerebral lateralization of cognitive function

**DOI:** 10.1016/j.jneumeth.2011.11.018

**Published:** 2012-03-15

**Authors:** Nicholas A. Badcock, Georgina Holt, Anneka Holden, Dorothy V.M. Bishop

**Affiliations:** a Department of Experimental Psychology, University of Oxford, Oxford, United Kingdom; b ARC Centre of Excellence in Cognition and its Disorders, Macquarie University, Sydney, Australia

**Keywords:** Functional transcranial Doppler ultrasonography, Imaging, Blood flow velocity, Methods, Matlab

## Abstract

We present a description of a new software package, ‘dopOSCCI’, which summarises data from experimental studies where functional transcranial Doppler ultrasonography (fTCD) is used to compare hemispheric rates of blood flow in order to assess lateralization of a cognitive process. The software provides a graphical user interface to summarise analogue and digital data collected using Multi-Dop Doppler Ultrasound devices (DWL Multidop T2: manufacturer, DWL Elektronische Systeme, Singen, Germany). The unique aspects of dopOSCCI allow multi-file processing, multi-event marker processing, behavioural and multi-session summaries, image file data visualization, and tab-delimited output files which includes split-half, single-trial summaries and data quality variables. The Matlab based software is available under the GNU GPL license and can be accessed online at https://databank.ora.ox.ac.uk/general/datasets/dopOSCCI, the Oxford University DataBank.

## Highlights

► We present a new functional transcranial Doppler ultrasongraphy toolbox for Matlab. ► The basic features include multi-file summaries and laterality index calculation. ► Advanced features include behavioural and multi-session summaries. ► The toolbox is freely available under the GNU GPL license.

## 1 Introduction

Functional transcranial Doppler ultrasonography (fTCD) is a non-invasive imaging technique which assesses changes in blood flow velocity in the cerebral arteries with high temporal resolution (Aaslid et al., 1982). While more complex techniques (e.g., functional Magnetic Resonance Imaging, fMRI or Magnetoencephalography) allow for relatively precise localization of activity, fTCD simply measures blood flow in a particular artery. One common application for fTCD is the assessment of language lateralization (for a review of applications see Stroobant and Vingerhoets, 2000), which may be for pre-surgical determination or psychological research. Functional lateralization is assessed by concurrent measurement of blood flow velocity in homologue cerebral arteries in the two hemispheres. Hemispheric lateralization of a cognitive domain is assumed if a greater increase in blood flow velocity can be reliably observed within one hemisphere during a domain specific task. The technique has good test–retest reliability (Knecht et al., 1998b; Stroobant and Vingerhoets, 2001) and provides results which are comparable to the Wada procedure (Knecht et al., 1998a; Knake et al., 2003) as well as fMRI (Knecht et al., 1999; Somers et al., 2011). Because fTCD is non-invasive, inexpensive, and portable, it is becoming more popular for psychological research comparing lateralization in special conditions such as Specific Language Impairment, autism, and dyslexia (Whitehouse and Bishop, 2008; Illingworth and Bishop, 2009), as well as children (Bishop et al., 2009; Stroobant et al., 2010; Haag et al., 2010). In this paper we report on a newly developed, open source, software package for processing fTCD data.

The standard fTCD paradigm for the assessment of language lateralisation is word generation introduced by Knecht et al. (1996,1998b), which has also been used successfully in fMRI (Benson et al., 1999). Participants are then asked to verbally report the generated words to confirm task compliance and following this, a rest period is required to return blood flow to baseline levels. During a series of such trials, Doppler probes, held in place at a position over the temporal window of the skull, insonate the middle cerebral arteries within the left and right hemispheres. Offline, the recorded blood flow velocity can be analysed to determine an individual's cerebral dominance for a particular task (for a detailed account see Ringelstein et al., 1990). Language tasks have also been developed for children (Lohmann et al., 2005; Bishop et al., 2009) and a video description of Bishop et al.’s technique is available (Bishop et al., 2010).

The basic processing paradigm was developed by Deppe et al. (1997) and is demonstrated in their software AVERAGE. Through a series of steps, AVERAGE normalizes the left and right signals, marks potential artefacts, integrates variation due to the cardiac cycle, adjusts for baseline activity, and averages the resulting signals over the series of suitable epochs. Using these averages, a difference signal is calculated as the left minus right signals. Within a specified period of interest, the peak difference is located. The sum of the left minus right difference over a 2 s period surrounding this peak is taken as an estimate of lateralization or the laterality index (LI). Positive numbers correspond to leftward lateralization and negative numbers to rightward.

**Fig. 1:**
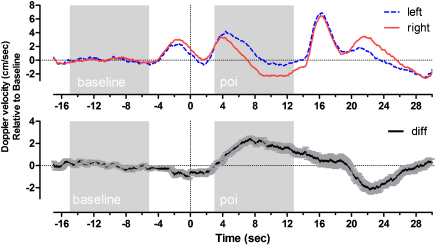
Example data for a fTCD word generation task. Upper panel displays the baseline corrected left and right Doppler velocity signals and the lower panel displays the left minus right difference (with standard error bars) as a function of trial time in seconds. Time zero indicates the presentation of the stimulus letter. Baseline and period of interest (poi) intervals are indicated by shaded sections. These data are the averages from 22 adults created using a dopOSCCI output file in Prism (GraphPad Software Inc., La Jolla, CA, USA). Four characteristic peaks are evident in the left and right signals (upper panel). These reflect (1) a generalized task preparation (to a ‘Ready’ signal), (2) a silent word generation at which point the left signal is greater than the right (language processes), (3) verbal report, and (4) the start of relaxation at which point the right signal is greater (potentially reflecting suppression of the verbal material).

Example left, right, and difference signals for the word generation task in 22 adults are presented in Fig. 1. During the period of interest (POI; i.e., the silent word generation) relatively higher cerebral blood flow velocity in the left compared to the right artery can be seen (Fig. 1, upper panel). This indicates leftward dominance, typical of participants engaging in a language task. As can be seen in the lower panel of Fig. 1, the standard error of the averaged blood flow is small, not overlapping with zero, indicating that the changes in blood flow were reliable across task repetitions and participants.

### 1.1 Introducing dopOSCCI

dopOSCCI has been developed to simplify the analysis of fTCD data collected using two probes in an event-related experimental procedure. The major advantages include multi-file processing (Section 2.2), multi-event marker processing (Section 2.4.3), behavioural (Section 2.6.3) and multi-session (Section 2.6.4) summaries, image file data visualization (Section 3.1), and tab-delimited output files which includes split-half, single-trial summaries (Section 3.2.2), and data quality variables (Section 3.2.3).

## 2 Materials and methods

We have developed a Matlab (Mathworks, Natick, MA, USA) based software package called ‘dopOSCCI’. The software includes a series of Matlab functions as well as a user-friendly graphical user interface. All necessary functionality can be achieved through the interface; therefore, knowledge of Matlab is not necessary to use the software.^1^

### 2.1 Data processing

The basic data processing steps are based upon Michael Deppe's work (Deppe et al., 1997, 2004a; Knecht et al., 2001) and personal correspondence.

### 2.2 File selection

**Fig. 2:**
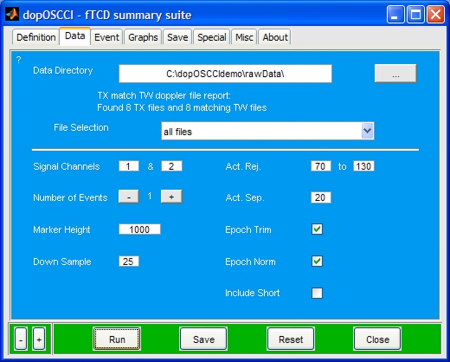
dopOSCCI Data tab of the graphical user interface. This tab allows for the specification of data directory, file selection, signal channels of left and right blood flow velocity data in data file, the number of events, marker height, down sample Hertz, activation rejection range, and acceptable activation separation, as well as the choice of epoch trimming, epoch normalization, and inclusion of data short epoch in the final summaries.

dopOSCCI handles files from two different software packages: Multi-flow (Multi-dop X4 by DWL Elektronische Systeme GmbH) and QL Monitoring Software (DWL, Compumedics Germany GmbH). Two of the files created by the Multi-Flow software are processed in dopOSCCI: ‘.TX’ and ‘.TW’. ‘.TX’ files hold session statistics whereas ‘.TW’ files hold blood flow velocity recordings as well as external channels inputs. Recordings made using the QL Monitoring Software are saved as ‘.EXP’ which include session information in the headers of the file along with left and right blood flow velocity, a series of optional outputs, and, when included, an analogue event maker. Raw data files are summarised and results collated using batch processing so that a collection of files are processed in a single step. It is also possible to select an individual file from a folder list which can be useful for data inspection and exploration of parameter settings. The data directory specification and file selection, as well as the data handling specification options as available in the graphical user interface can be seen in Fig. 2.

### 2.3 Data handling specifications

The dopOSCCI Data tab (see Fig. 2) houses a series of options that control the initial handling of the raw data including import, normalization, and unacceptable signal detection.

#### 2.3.1 Signal channels

The raw data output of the analogue Doppler systems is typically organised with the left and right blood flow velocity saved in channels 1 and 2 (the default dopOSCCI settings). The newer digital systems commonly default to columns 3 and 4; therefore, specification of this information can be made.

#### 2.3.2 Event markers

For the purpose of fTCD, cues are required to be stored in the data file in order to time-lock the task-related activity. These are commonly sent via the parallel port. The Doppler hardware allows for the specification of the height and width of these markers or pulses; however, the dopOSCCI method for detecting these markers simply records the onset of a marker of any width, above a specified marker height setting. This is, anything above the specified value will be treated as a marker and anything below that value will be ignored. As for the signal channels, it is critical to specify the location of the event channel in the raw output file. This is done on the Event tab (see below).

#### 2.3.3 Down sampling

The Doppler hardware is capable of recording high frequency data, sometimes recording velocities 100 times per second (i.e., 100 Hz). This results in samples every 10 ms which may be considered unnecessary for assessing a response in the range of 5–10 s (Aaslid, 1987; Rosengarten et al., 2002). For this and historical computational power limitations, the data is downsampled which involves maintaining a reduced number of the available samples. For example, if the data were recorded at 100 Hz and are to be downsampled to 25 Hz, then every 4th sample is maintained in the analysis and all others dropped.

#### 2.3.4 Normalization

Due to potential difference in the recorded left and right blood flow velocities arising from measurement artefacts such as probe angle (see Deppe et al., 2004b), i.e., the signal for the left may be quantitatively higher than the right, the left and right data are normalized to a mean of 100 using the following equation (100 × data)/mean (data): where data refers to a collection of blood flow velocities values. These values could be for the entire left or right recordings or from subsections of these recordings (see Section 2.3.5). The formula shifts the average level of the signal while maintaining the variance.

#### 2.3.5 Data trim and epoch normalization

At the beginning, rest breaks, or end of an fTCD task, there is often wide variation in the recorded activity due to participant movement. Rather than include this activity in the normalization of the data, dopOSCCI provides the Data Trim option. This option removes data preceding and following that specified by the event epoch settings (see below) before the normalization adjustment.

Across an experimental session, the probe position may be modified either through gross movements made by participants, adjustments made by the experimenter in response to participant movements or general signal changes, or signal loss due to probe ‘drift’, i.e., gradual, subtle movement of the probe. In some cases such changes are difficult to monitor in an online testing environment, especially with children, and are therefore only evident at the analysis stage. At worst, drift can bias LI calculation on a trial by trial basis. Normalizing the data by epoch removes these artefacts, setting the left and right values to equivalent levels within each epoch based upon event timings (see Section 2.4.2).

#### 2.3.6 Heart cycle exclusion

One major physiological activity that affects the measured blood flow velocity is the involuntary cardiac cycle. This variation interferes with examination of the task-related signal. An elegant correction for this variation is to take an average of the activity within a single heart cycle, resulting in a step-like summary of the activity as opposed to a widely varying heart beat (Deppe et al., 1997). This technique is employed in dopOSCCI.

#### 2.3.7 Epoch rejection

Following normalization, the data within each epoch are screened for variation due to measurement artefact. dopOSCCI considers two forms of this variation: range and signal separation. With respect to the range, epochs with left or right signal values outside the set upper and lower acceptable values can be excluded from later stages of analysis. Epochs with values less than or greater than 30–50% of mean activation are commonly excluded.

With respect to left–right signal separation, in certain circumstances a single channel may be disrupted resulting in a substantial drop in signal level; however, this drop does not result in values outside the lower limit of the activation rejection range. Setting the acceptable activation separation creates an upper limit on the effect of these disturbances.

### 2.4 Event specification

**Fig. 3:**
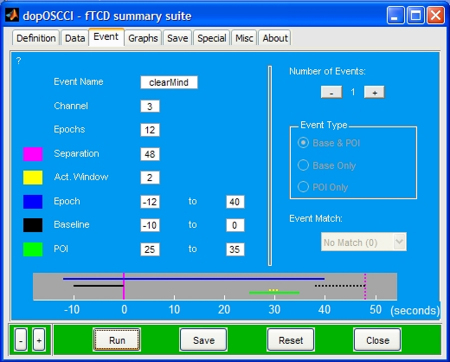
dopOSCCI Event tab of the graphical user interface. This tab allows for the specification of event related information including the event name, data channel or column in data file, the number of epochs, the temporal separation between epoch markers, the length of the activation window, the temporal position of the epoch, baseline, and period of interest settings with respect to the epoch markers.

The dopOSCCI Event tab (see Fig. 3) houses a series of options to specify the event details; most notably, the event timings for epoch, baseline, and period of interest (POI) intervals.

#### 2.4.1 Epoch definition: number and separation

dopOSCCI searches for a specified number of epochs separated by a specified time interval. It assumes the final marker is a true marker and examines the temporal difference between markers in reverse order; i.e., from the last marker working towards the first marker. Event markers that occur at less than the specified separation, e.g., 50 s, will be ignored in favour of the next marker. The process continues until the specified number of epochs is found or the available number of event markers has been examined. This process is useful in dealing with spurious event markers which are sometimes found in fTCD files. These may occur through the sending of markers during a practice phase of the task or through hardware communication error. In our experience extraneous markers occur at the beginning of the data files, therefore examining the markers in reverse order overcomes is issue.

#### 2.4.2 Event timings

Upper and lower values for epoch, baseline, and period of interest timings should be specified with respect to the event marker which is set as time 0. Using the example of the word generation task, if the event marker reflects the onset of the letter, then the epoch could be set from −20 to 30 s; baseline −15 to −5; and period of interest, 3 to 13. This epoch period encapsulates 55 s of the 60 s trial, includes 5 s before the baseline begins, and 17 s after the period of interest. This epoch also dictates the information which will be displayed in any graphs or average activation data (see Section 3). This baseline period includes 10 s of activation at the end of the long rest period and this period of interest includes a 10 s period across which silent word generation activity can be monitored. It is within the period of interest that the peak left–right activity difference is examined in order to calculate the LI. Also specified on the Event tab is the Activation Window which indicates the time across which the LI will be calculated, usually 2 s.

#### 2.4.3 Multiple events

dopOSCCI includes an option to summarise data with respect to multiple event markers. If using a single event marker, baseline and period of interest timings are set relative to a single event marker; e.g., baseline = −10 to −2 s (before the event marker), period of interest = 15–25 s (after the event marker). If using two or more event markers, baseline and period of interest timings can be locked to each marker. This is useful if two or more experimental conditions are presented. Using this system, different timings can be set for each event marker.

Alternatively, if the time interval between the baseline and period of interest is variable, the baseline timings can be set relative to event marker 1 and the period of interest timings can be set relative to event marker 2. These circumstances would occur when the period of interest is locked to a participant's response which could vary between epochs.

### 2.5 Baseline correction

There are four spontaneous physiological processes that influence cerebral blood flow velocity. Correcting for the influence of the cardiac cycle removes the highest frequency effects but in order to remove lower-frequency effects, which include breathing and less understood variations in sympathetic system activity and states of arousal (Deppe et al., 2004b), baseline correction is performed on an epoch by epoch basis (Deppe et al., 1997). For the left and right activity, average activation during the baseline period is subtracted from all other activity within the epoch. Therefore, deviations from zero reflect increases or decreases in activity relative to baseline. dopOSCCI performs a large array of calculations, the most critical for fTCD being the LI, and all calculations are performed on the baseline corrected data.

### 2.6 Special functions

In addition, dopOSCCI also provides four special functions that we have found useful for data processing. These include manual epoch screening, group summaries, behavioural summaries, and combining multiple sessions.

#### 2.6.1 Manual epoch screening

Manual epoch screening allows for the selective exclusion of epochs for each individual data file. For example, during a testing session, if a participant was clearly not engaged in the task during particular epochs, these can be excluded from the set of epochs used to calculate the LI.

#### 2.6.2 Group summaries

Group summaries categorize the data files into manually selected groups. This is useful for early visualization for group comparisons as well as later stages of data analysis, especially when contrasting typical versus atypical groups. dopOSCCI also includes the grouping variable in the output files, which can be used for further analysis.

#### 2.6.3 Behavioural summaries

The behavioural function produces summaries based upon selections of epochs within individuals. We have two approaches which use this function. The most straight forward is an experimental condition summary. For example, in a visual land-mark task we manipulated difficulty in three conditions (unpublished data of our group). These conditions were presented in a different random order to each participant and using the dopOSCCI behavioural function, we summarised the data in collections of trials grouped by condition. Another example is data-driven categorisation based upon participants’ responses (Badcock et al., in press). In a standard word generation paradigm (described above) we categorized letters with respect to the number of words verbally produced. We created three levels of word production based upon behavioural responses of the group and then summarised epochs for each individual based on these categorizations.

#### 2.6.4 Session summaries

In some cases individual participants have completed multiple sessions (Groen et al., 2011). For example, completing a task first with the left and then with the right hand. dopOSCCI provides a function to combine this data, first summarising each separately and then in combination, providing an output which can be easily interrogated to determine session differences and look at the overall patterns, independent of session.

## 3 Results and discussion

### 3.1 Data visualization

**Fig. 4:**
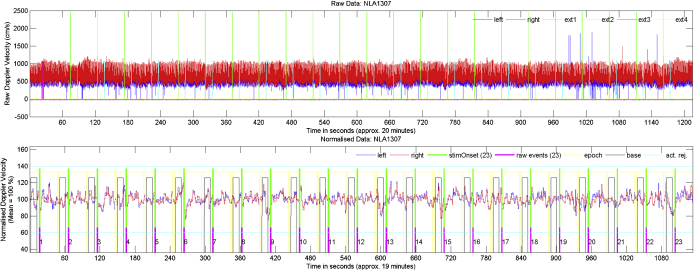
Example raw (upper panel) and normalized (lower panel) graphs produced by dopOSCCI. These are based upon word generation data from a single individual. The raw graph displays the raw left and right blood flow velocity recordings as well as any event markers recorded in the external channels. The normalized graph displays the left and right blood flow velocity after basic data processing (described in Section 2.3). This graph also displays event markers, epoch and baseline divisions, and activation rejection limits.

**Fig. 5:**
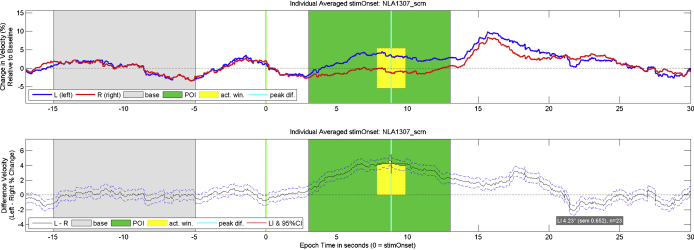
Example of an average graph produced by dopOSCCI. These are based upon word generation data from a single individual, averaged across 23 trials. The upper panel displays the left and right baseline corrected percentage change in blood flow velocity and the lower panel displays the difference between the left and right baseline corrected blood flow velocities. The baseline runs from −15 to −5 s relative to the visual letter presentation, and the period of interest (POI) runs from 3 to 13 s following the visual letter presentation. The time point of the peak difference is displayed in both panels, surrounded by a 2-s activation window across which the laterality index (LI) is calculated. In the lower panel, the 95% confidence interval of the LI calculations is also displayed along with the LI value, the LI standard error of the mean, and the number of epochs averaged within the graph.

dopOSCCI provides three types of graphs for data visualization which can be viewed during the processing or saved for later inspection (see Figs. 4 and 5 for examples of these graphs). The graphs can be saved in a variety of image formats (e.g., jpeg, tiff, bmp) depending upon particular needs; e.g., Matlab's ‘.mat’ format is available which can be opened in Matlab for modification. The three types of graph include (1) raw data which displays the raw left and right signals and event markers, (2) matched data which displays normalized left and right signals, raw and to-be-used event markers, baseline and epoch intervals, and activity rejection range, and (3) average data which displays the epoch averaged data for the baseline corrected left, right, and difference signals (see Fig. 5). Average data graphs can be created for an individual's accepted epochs, each single epoch, behavioural summaries (see Section 2.6.3), and all processed data (an overall average based upon multiple files).

### 3.2 Data output

Individual results are collated in tab-delimited text files which can be easily imported into programs such as SPSS, Excel, or R. In addition to a series of output variables (see Section 3.2.1) graphing data is included in a separate output file. The graphing data includes left, right, left minus right difference, and left–right average activation across the specified epoch period. This data reflects individual activation, averaged across accepted epochs.

#### 3.2.1 Summary variables

A series of summary variables is available by selection. These include traditional LI indices based upon the mean left minus right difference at the point of the maximum difference within a period of interest. Additional variables are also available. These can be based upon independent left or right signals as well as the left minus right difference or the left–right average signals. The available descriptive statistics include the mean, standard deviation, standard error of the mean, 95% confidence interval, minimum, maximum, root mean square, median, and inter-quartile range. These variables can be calculated with respect to fixed periods such as the baseline or period of interest as well as surrounding calculated periods such as the maximum or minimum value within a fixed period. For example, one could extract the maximum left–right averaged signal within the period of interest in order to determine the point at which the overall haemodynamic response was greatest. The array of summary variables provides a large degree of flexibility for the exploration of Doppler data. Further to this, the significance of the LI coefficients can be examined using parametric and non-parametric, single- and two-sample inferential tests. For single-sample tests, LI values are examined against 0, and for the two-sample tests, left and right averages are compared. Inferential outputs include test statistics, *p*-values, and effect sizes.

For the purposes of demonstrating a few of the summary variables, the processing was run on the data from a single participant completing the word generation task (displayed visually in Fig. 5). The LI for the 23 averaged trials is 4.23 (SD = 3.13, Cohen's *d* = 1.23), reflecting significant left lateralization. Calculations based upon the average of the left and right signals indicate that activation peaked at 17.68 s after visual letter presentation (i.e., during verbal report) with an average change in blood flow velocity of 7.68% (SD = 2.31) of baseline blood flow velocity. Further to this, each of these calculations can also be performed for each epoch; for example, the LI range calculated for individual epochs is −6.12 to 10.9. This reflects a large variation which can be readily explored by examining graphs for individual epochs.

#### 3.2.2 Task reliability calculations

The internal reliability of fTCD results can be estimated by calculating the LI for the odd and even epochs and correlating these within a set of group results: the split-half reliability (as in Bishop et al., 2009). In dopOSCCI, additional options are available to output the LIs for odd versus even epochs as well as a random half-split of epochs. However, a more accurate estimate of internal reliability is Cronbach's alpha (Cronbach, 1957) which uses all split-half iterations to provide an estimate of reliability. An optional dopOSCCI output provides the LI for each epoch (as used in Whitehouse et al., 2009) which can be used to calculate Cronbach's alpha.

#### 3.2.3 Data quality variables

Maintaining suitable left and right fTCD signals with adults can be difficult. However, the biggest challenge we have experienced has been with children who tend to be more active which often disrupts the signal. In order to assess this disruption an optional dopOSCCI output is signal dropout. This can be used to exclude individual data or epochs.

The second data quality variable available in dopOSCCI is the Goodness of Recording assessment introduced by Knecht et al. (2001). This statistic provides an estimate of the variability (root mean square) in the baseline period which can be compared against a criterion to exclude individual data files. Knecht et al. suggest left or right signal variation greater than 2% of average baseline activity (normalized to 100) as a criterion for data exclusion.

## 4 Conclusion

The use of fTCD to assess cerebral dominance of cognitive functions is becoming increasingly popular and therefore, developing tools to simplify the processing will be useful. We have described a new software package, dopOSCCI, which summarises Doppler data in an efficient manner allowing researchers to visualize and interrogate data through new and innovative methods. The software is open source and can be accessed online at https://databank.ora.ox.ac.uk/general/datasets/dopOSCCI, the Oxford University DataBank.

## Footnotes

1 dopOSCCI does operate at the command line. However, unlike the flexibility enabled through toolboxes like EEGLAB (Delorme and Makeig, 2004), due to the encapsulated nature of current fTCD analysis, function level operations add little to the analysis other than low-level data exploration.
